# Temporal trends in stillbirth over eight decades in England and Wales: A longitudinal analysis of over 56 million births and lives saved by improvements in maternity care

**DOI:** 10.7189/jogh.12.04072

**Published:** 2022-09-17

**Authors:** Gbenga A Kayode, Andrew Judge, Christy Burden, Cathy Winter, Tim Draycott, Basky Thilaganathan, Erik Lenguerrand, Tommy’s National Centre for Maternity Improvement

**Affiliations:** 1 Translational Health Science, Bristol Medical School, University of Bristol, Southmead Hospital, Bristol, United Kingdom; 2 Royal College of Midwives, London, United Kingdom; 3 The PROMPT Maternity Foundation, Department of Women's Health, Southmead Hospital, Bristol, United Kingdom; 4 Royal College of Obstetricians and Gynaecologists, London, United Kingdom; 5 St. George’s University Hospitals, London, United Kingdom

## Abstract

**Background:**

Considering the public health importance of stillbirth, this study quantified the trends in stillbirths over eight decades in England and Wales.

**Methods:**

This longitudinal study utilized the publicly available aggregated data from the Office for National Statistics that captured maternity information for babies delivered in England and Wales from 1940 to 2019. We computed the trends in stillbirth with the associated incidence risk difference, incidence risk ratio, and extra lives saved per decade.

**Results:**

From 1940-2019, 56 906 273 births were reported. The stillbirth rate declined (85%) drastically up to the early 1980s. In the initial five decades, the estimated number of deaths per decade further decreased by 67 765 (9.49/1000 births) in 1940-1949, 2569 (0.08/1000 births) in 1950-1959, 9121 (3.50/1000 births) in 1960-1969, 15 262 (2.31/1000 births) in 1970-1979, and 10 284 (1.57/1000 births) in 1980-1989. However, the stillbirth rate increased by an additional 3850 (0.58/1000 births) stillbirths in 1990-1999 and 693 (0.11/1000 births) stillbirths in 2000-2009. The stillbirth rate declined again during 2010-2019, with 3714 fewer stillbirths (0.54/1000 births). The incidence of maternal age <20 years reduced over time, but pregnancy among older women (>35 years) increased.

**Conclusions:**

The stillbirth rate declined drastically, but the rate of decline slowed in the last three decades. Though teenage pregnancy (<20 years) had reduced, the prevalence of women with a higher risk of stillbirth may have risen due to an increase in advanced maternal age. Improved, more personalised care is required to reduce the stillbirth rate further.

Stillbirth is a neglected global tragedy, with over 7000 deaths daily worldwide [1]. Although stillbirths occur predominantly in low- and middle-income countries (LMICs), about one stillbirth occurs for every 200 births in high-income country (HIC) settings [2]. The impact of stillbirth is debilitating, with long-lasting psychological and social consequences on mothers and their families [3]. Stillbirth rate remains an important indicator of the quality of antenatal care, which is principally focussed on reducing preventable foetal loss. Congruent with other HICs, stillbirth remains an important public health issue in the United Kingdom (UK), with a clinical burden two times higher than that of neonatal death [4,5]. The stillbirth rates in HICs had declined drastically for the five decades preceding the 1990s [6,7]. The interventions that contributed to the rapid decrease in stillbirth rates over this period appear to have impacted intrapartum stillbirths more than antepartum stillbirths [4,8]. Consequently, the risk of antepartum stillbirth is up to 10-fold higher than intrapartum stillbirth [4,8].

Multiple interventions have been implemented to reduce the risk of stillbirth in the UK, including the ambitious target of the UK Department of Health and Social Care to reduce the stillbirth rate by half before 2025 [9,10]. Accurate measurement is a key part of improvement and recent studies examining the trends in stillbirth in the UK and other settings have only depicted the trends in stillbirth graphically and confined the analysis to the annual rate of stillbirth [6,7,11]. The latter types of analysis can lead to overly simplistic conclusions, as well as difficulties with interpretation by stakeholders. The aim of this study is to quantify the risk of stillbirth over time, assess sex disparity in stillbirth [12], and present the stillbirth data as lives saved over time to make the results comprehensible as much as possible to all stakeholders.

## METHODS

### Study design

This population-based longitudinal study examined the trends in stillbirths among total births in England and Wales from January 1, 1940, to December 31, 2019.

### Data sources

This study utilized publicly accessible, aggregated maternity information on babies delivered in England and Wales, published by the Office for National Statistics (ONS).[13] The available data captured were limited to birth year, total births, total stillbirths, baby sex, maternal age, and marital status. Maternal age was categorized as <20 years, 20-29 years, 30-34 years, 35-39 years, and ≥40 years. Marital status was classified as within marriage (or civil partnership) and outside marriage (or civil partnership).

### Study population

Participants included in the data were babies born in England and Wales from January 1, 1940, to December 31, 2019.

### Outcome

Stillbirth was defined as a baby born at or after 24 weeks gestational age with no sign of life.

### Statistical analysis

Participants’ characteristics were expressed as frequencies and percentages for categorical data. Non-normally distributed continuous data were presented as medians and interquartile range (IQR). To visualise changes in stillbirth over eight decades, we computed the trends in stillbirths from 1940 to 2019 in England and Wales using the aggregated ONS data. Subsequently, sex-specific trends in stillbirths were generated over the same period to examine sex disparity. To capture the actual public-health consequences of the trends in stillbirth and accurately quantify the relative impact of the implemented interventions, we estimated the incidence risk difference (IRD) and incidence risk ratio (IRR) with their 95% confidence intervals (95% CI) by comparing the risk of stillbirth at the beginning and end of each decade. Finally, we estimated the total number of babies saved due to the change in stillbirth rate per decade. This is the first study to describe trends in stillbirth rate in terms of number of lives saved.

### Ethics and permission

This study was conducted using publicly accessible ONS data. No ethical approval was required.

## RESULTS

### Participant characteristics

From January 1, 1940, to December 31, 2019, a total of 55 670 590 births (median = 682 922.5 per annum; interquartile range (IQR) = 84 084.5) and 717 908 stillbirths (median = 4940.5 per annum; IQR = 12 167.5) were reported. The incidence of maternal age <20 years increased from 3.7% in the 1940s and peaked in 1970-79 (10.3%), then reduced to 3.9% in 2010-2019. Conversely, maternal age >35 years (age group 35-39 years and ≥40 years) reduced from 17.6% in 1940-1949 to 6.4% 1970-1979, then increased to 21.4% in 2010-2019. The proportion of women married or in a civil partnership fell from 95.2% in 1950-1959 to 52.3% in 2010-2019. (**Table 1**).

**Table 1:** Characteristics of women who delivered in England and Wales from 1940 to 2019

Year	Number of mothers	Maternal age at delivery (years)						Marital status
**≤20**	**20 - 24**	**25 - 29**	**30 - 34**	**35 - 39**	**≥40**	**Married**
1940-1949	7 144 032	3.7%	25.7%	30.5%	22.5%	13.3%	4.3%	93.9%
1950-1959	6 987 127	5.1%	28.8%	31.8%	20.6%	10.5%	3.2%	95.2%
1960-1969	8 326 571	8.9%	32.9%	30.3%	17.1%	8.3%	2.5%	92.7%
1970-1979	6 600 339	10.3%	33.0%	34.8%	15.5%	5.1%	1.3%	90.9%
1980-1989	6 562 857	8.6%	29.4%	34.5%	19.8%	6.5%	1.2%	81.0%
1990-1999	6 631 607	7.2%	21.2%	34.4%	26.6%	9.9%	1.7%	66.3%
2000-2009	6 476 795	6.9%	18.8%	26.3%	28.9%	15.8%	3.3%	57.3%
2010-2019	6 941 172	3.9%	16.2%	27.9%	30.6%	17.2%	4.2%	52.3%

### Temporal trends in stillbirths

**Figure 1:**
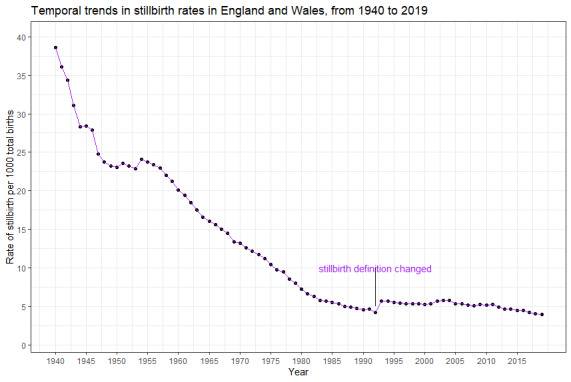
Temporal trends in stillbirth rates in England and Wales, from 1940 to 2019.

**Figure 2:**
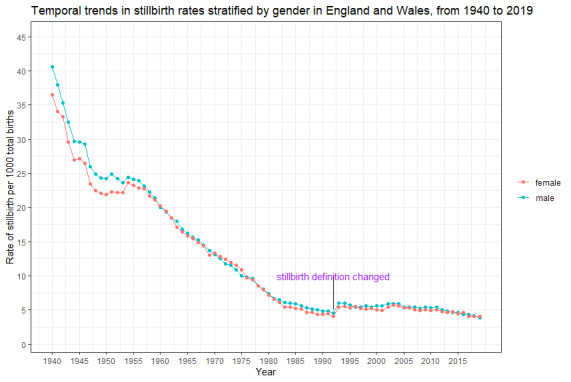
Temporal trends in stillbirth rates stratified by gender in England and Wales, from 1940 to 2019.

**Figure 1** demonstrates the trends in stillbirth in England and Wales from 1940 to 2019. There was a 39.9% decline in stillbirths from 38.6 per 1000 births in 1940 to 23.2 per 1000 births in 1949. The stillbirth rate remains unchanged until 1954 when it declined steadily from 24.0 per 1000 births to 4.3 per 1000 births in 1992, a reduction of 82%. In the last three decades, the stillbirth rate remained nearly stagnant in the early part of this era, but later reduced by 31.2% during 2010-2019. The sex-specific trends in the stillbirth rate are presented in **Figure 2**. Although the stillbirth rate in males appeared slightly higher than that of the female until 1957, the sex disparities disappeared thereafter as the estimates started overlapping.

### Changes in the incidence rate difference (IRD) and incidence rate ratio of stillbirth (IRR)

Results of the estimated IRD and IRR of stillbirth per decade are presented in **Table 2**. From 1940 to 1949, a reduction of 15.4 per 1000 births (95% CI = -16.01, -14.79) was observed in the incidence rate of stillbirth, ie, a relative reduction of 40% (95% CI = 39%, 41%). Between 1950 and 1989, the risk of stillbirth fell by 8% (95% CI = 6%, 10%), 34% (95% CI = 32%, 35%), 39% (95% CI = 37%, 41%) and 35% (95% CI = 32%, 38%) in each decade, respectively. Unlike other previous decades, the incidence rate of stillbirth increased by 0.70 per 1000 births in the 1990s, corresponding to a 15% relative increase (95% CI = 10%, 21%). In the following decade (2000 to 2009), the rate of stillbirth remained statistically unchanged followed on by a decrease of 23% in the risk of stillbirth (95% CI = 19%, 27%) from 2010 to 2019.

**Table 2:** Incidence rates, differences, and rates ratio of stillbirth per 1000 total births per decade from 1940 to 2019

Year	Total births	Stillbirths	Beginning of decade	End of decade	IRD (95% CI)*	IRR (95% CI)†
1940-1949	7 144 032	207 995	38.60	23.20	-15.40 (-16.01, -14.79)	0.60 (0.59, 0.61)
1950-1959	6 987 127	160 624	23.07	21.24	-1.83 (-2.32, -1.34)	0.92 (0.90, 0.94)
1960-1969	8 326 571	138 659	20.15	13.36	-6.79 (-7.20, -6.40)	0.66 (0.65, 0.68)
1970-1979	6 600 339	71 796	13.19	8.03	-5.15 (-5.49, -4.82)	0.61 (0.59, 0.63)
1980-1989	6 562 857	37 428	7.27	4.71	-2.57 (-2.83, -2.31)	0.65 (0.62, 0.68)
1990-1999	6 631 697	34 422	4.61	5.31	+0.70 (+0.46, +0.94)	1.15 (1.10, 1.21)
2000-2009	6 476 795	35 020	5.30	5.22	-0.08 (-0.33, +0.17)	0.99 (0.94, 1.03)
2010-2019	6 941 172	31 964	5.14	3.94	-1.20 (-1.42, -0.97)	0.77 (0.73, 0.81)

IRD – incidence rate difference, IRR – incidence rate ratio, CI – confidence interval

*Incidence rate of stillbirth at the end of a decade – incidence rate of stillbirth at the beginning of a decade.

†Incidence rate of stillbirth at the end of a decade/incidence rate of stillbirth at the beginning of a decade.

### Extra lives saved per decade

**Table 3** presents the clinical impact of the observed changes in stillbirth rate per decade expressed as additional lives saved. The reduction in stillbirth rate between 1940 and 1949 saved 67 765 lives and prevented an additional 9.49 stillbirths per 1000 births. Between 1950 and 1989, the observed reduction in stillbirth resulted in 569 lives (1950-1959), 29 121 lives (1960-1969), 15 262 lives (1970-1979) and 10 284 lives (1980-1989) saved in each decade. In the subsequent two decades (1990-2009), 3850 and 693 more stillbirths were reported. In 2010-2019, the decline stillbirth rate saved 3714 lives, and prevented 0.54 stillbirths per 1000 births.

**Table 3:** Additional lives saved per decade due to change in stillbirth rate

Year	Observed live births*	Expected live births†	Extra lives saved/lost‡	Extra lives saved/lost per 1000 births§
1940-1949	6 936 037	6 868 273	+67 765	+9.49
1950-1959	6 826 503	6 825 934	+569	+0.08
1960-1969	8 187 912	8 158 791	+29 121	+3.50
1970-1979	6 528 543	6 513 281	+15 262	+2.31
1980-1989	6 525 429	6 515 145	+10 284	+1.57
1990-1999	6 597 275	6 601 125	-3850	-0.58
2000-2009	6 441 775	6 442 468	-693	-0.11
2010-2019	6 909 208	6 905 495	+3714	+0.54

*Total live births per decade as in the data.

†Total births per decade – (total births per decade × stillbirth rate at the beginning of the decade).

‡Additional number of lives saved (+) or lost (-) due to the decline (+) or increase (-) in stillbirth rate over a decade = observed live births – expected live births.

§Lives saved per 1000 births = additional number of lives saved (+) or lost (-) per 1000 births due to the change in stillbirth rate over the decade of interest.

## DISCUSSION

This longitudinal study examined stillbirth rates over eight decades. The annual rate of decline of 0.12 per 1000 births in the last decade (2010-2019) is modest compared with a reduction in stillbirth of 1.54 per 1000 births observed from 1940-1949. There was a decrease in the number and rate of stillbirths for all except two decades (1990-2009). There was a parallel increase in maternal age as well as in the proportion of women who were not married or in civil partnerships.

The change in stillbirth observed in this study is consistent with the patterns observed in other HICs, including Sweden, France, Italy, Netherlands, Spain, and the United States [6,14]. The period of rapid reduction in stillbirth rate that started in the 1940s has been attributed to the improvements in maternal care services delivered by birth attendants, routine use of antisepsis measures, and availability of new drugs for infection control [2,15-17]. Besides, the observed changes in the trends of stillbirths were outcomes of complex interactions between multiple interventions, health policies, and patient- and contextual-level determinants of stillbirth. Nevertheless, further work is still required to understand and tackle this challenge. The interventions implemented during this era were not specifically targeted at reducing stillbirth, but were intended to improve maternal health and well-being translating into improved pregnancy outcomes more broadly [18].

In the last three decades, the reduction in stillbirth rate has slowed in England and Wales. This finding is consistent with other HICs [6,14,19,20], and better than some settings where the stillbirth rate has principally stalled or increased [21,22]. The recent modest improvement observed in stillbirth in HICs has previously been shown to occur mainly among late gestational stillbirths (28 weeks and above) [2,23]. This may be attributed to improved perinatal diagnosis and advanced foetal, neonatal, and maternal care [24-26]. Some key interventions and health policies that were implemented in the UK to improve pregnancy outcomes include (but are not limited to) the following: the Department of Health & Social Care’s National Maternity Safety Strategy ambition to half the rate of stillbirth and reduce preterm births from 8% to 6% [9,10], NHS England’s Maternity Transformation Programme [27], National Maternity Review Better Births [28], the Royal College of Midwives (RCM)’ Better Births programme [29], NHS England’s Saving Babies’ Lives Care Bundle [30], the Royal College of Obstetricians & Gynaecologists(RCOG) Each Baby Counts national quality improvement programme [31], NHS Improvement’s Maternal and Neonatal Health Safety Collaborative, a three-year programme [32], the National Maternal & Perinatal Audit [33], Maternity and Children Quality Improvement Collaborative [34].

The recent slow rate of decline in stillbirth in HICs may be related to multiple factors, including the quality of antenatal and intrapartum care [2], population increase in pregnancies at risk of stillbirth due to maternal comorbidities including diabetes, advanced maternal age, obesity, and inequality in perinatal health care related to socioeconomic deprivation [2,35,36], Also, the slow decline observed in stillbirth over the last three decades is in line with a common epidemiological phenomenon showing that risk prevention becomes challenging as the incidence reduces.

To bring the definition of stillbirth into alignment with concurrent improved gestational age of neonatal survival, the 1992 Stillbirth (Definition) Act was passed, reducing the gestational age threshold of stillbirth from 28 to 24 weeks’ gestation. This change to a more inclusive definition of stillbirth resulted in a statistical increase in stillbirth rate from 1990 to 1999 as demonstrated by IRD, IRR and lives saved. It is important to understand that stillbirth definitions vary between countries and that these differences limit or influence direct inter-country comparisons of stillbirth rates [37].

In 2015, the UK Department of Health proposed to reduce stillbirth estimated at 3.87 per 1000 births in 2015 by half before 2025 [38,39]. Based on our findings, this ambition could only be achieved in England and Wales with a minimum annual rate of decline of 0.23 stillbirths per 1000 births. The current annual decline of 0.14 per 1000 births is insufficient to meet these targets and the actualization of this goal is likely to be prolonged beyond 2030 in England and Wales. As with many other HICs, there has been an increase in pregnancies at risk of placental dysfunction and stillbirth, with advanced maternal age [40,41], obesity, diabetes and heart disease all being recognised risk factors for stillbirth (among others) [42]. The current system for assessing risk of placental dysfunction and stillbirth in pregnancy based on the presence or absence of population-based risk factors in a checklist approach is neither accurate nor effective [43,44]. A more personalised care approach to such risks assessment which takes in the absolute risk of each factor and their inter-relationship and produces a numerical risk algorithm to allow personalisation and triage of antenatal investigations and care provision is long overdue.

This study analysed reliable, longitudinal official data on all births in England and Wales. To date, this is the first study in this setting to formally assess the actual burden of stillbirth over time and accurately quantify the relative impact of simultaneous interventions implemented. The analysis was presented as lives saved to improve accessibility of the results for all stakeholders. Nevertheless, this study has some limitations. First, no causal relationship can be established from this study. Also, there were few characteristics of the mothers and babies included in the ONS data; in particular, the ethnicity of the mother and parents are unavailable. Ethnicity and other inequality-related factors need to be explored to examine the existing inequalities in the stillbirths over decades. The observed early decreases in stillbirth rates are likely to be larger considering the uncertainty surrounding the completeness of the data in the 40s to early 60s.

## CONCLUSIONS

This study demonstrated that stillbirth had declined significantly in England and Wales since 1940. However, in the last three decades, the reduction in stillbirth rate has been modest despite increased awareness and national initiatives to reduce the rate. As the population of women at risk of stillbirth continues to increase despite the ongoing effort to address stillbirth, it is necessary to consider the introduction of personalised risk assessment in pregnancy to permit effective triage to appropriate levels of assessment and prompt interventions in pregnancies at higher risk of placental dysfunction, foetal growth restriction and stillbirth.
